# Columnar Architecture Improves Noise Robustness in a Model Cortical Network

**DOI:** 10.1371/journal.pone.0119072

**Published:** 2015-03-17

**Authors:** Paul C. Bush, Zachary F. Mainen

**Affiliations:** Champalimaud Neuroscience Programme, Champalimaud Center for the Unknown, Lisbon, Portugal; Plymouth University, UNITED KINGDOM

## Abstract

Cortical columnar architecture was discovered decades ago yet there is no agreed upon explanation for its function. Indeed, some have suggested that it has no function, it is simply an epiphenomenon of developmental processes. To investigate this problem we have constructed a computer model of one square millimeter of layer 2/3 of the primary visual cortex (V1) of the cat. Model cells are connected according to data from recent paired cell studies, in particular the connection probability between pyramidal cells is inversely proportional both to the distance separating the cells and to the distance between the preferred parameters (features) of the cells. We find that these constraints, together with a columnar architecture, produce more tightly clustered populations of cells when compared to the random architecture seen in, for example, rodents. This causes the columnar network to converge more quickly and accurately on the pattern representing a particular stimulus in the presence of noise, suggesting that columnar connectivity functions to improve pattern recognition in cortical circuits. The model also suggests that synaptic failure, a phenomenon exhibited by weak synapses, may conserve metabolic resources by reducing transmitter release at these connections that do not contribute to network function.

## Introduction

Columnar architecture is a striking feature of neocortex characterized by similarity in the receptive field properties of cells encountered during a vertical penetration [1]. Neighboring cells tend to share similar parameter (feature) tuning [2] and this tuning varies continuously in the horizontal direction [3] at the single cell level [4], resulting in smooth maps punctuated by discrete jumps that are thought to be due to the constraint of fitting multiple parameter maps onto a two dimensional surface [5], [6]. Columnar architecture was discovered decades ago, yet there is still no agreement on its function. In fact, no differences between animals with and without columns have been found in single cell properties such as orientation tuning or behavioral metrics such as visual acuity [7]. This has led to the questioning of whether cortical columns have any functional role at all [8], [9].

Any form of topographic mapping could be considered a form of columnar architecture (retinotopy, barrel fields, etc.), derived from a mapping of the sensory surface that preserves existing order. However, columnar architecture is generally considered to apply only to parameters derived from intracortical processing, such as orientation and spatial frequency tuning [5], [8]. Interestingly, this form of columnar structure is not present in rodents [7], [10], [11] and likely evolved independently in carnivores and primates [12].

“Columns” were initially thought to be discrete structures [13] related to physical clustering of neuronal elements [5], as occurs in barrel fields [14]. However, a parameter such as orientation changes smoothly from one cell to the next [4], [11] with only occasional discrete jumps. With such continuous mapping, the choice of a center to define any single column is arbitrary. Therefore, rather than thinking of discrete computational modules, it is more useful to consider the significance of the local cortical connectivity of which the columnar architecture is a result.

The probability of connection between pyramidal cells in layer 2/3 of primary sensory cortex has recently been shown to be inversely proportional to the physical distance separating the cells [15], [16] and also inversely proportional to the distance between the preferred parameters of the cells [17].

Here, we use a computer model of 1 mm^2^ of layer 2/3 of cat primary visual cortex (V1) to explore the impact of columnar organization on cortical function. We simulate the local connectivity within the area of one hypercolumn, the minimal size needed to demonstrate the effects of columnar connectivity. We find that the interplay between columnar organization (parameter mapping) and the experimentally-observed dependence of connection probability on the distance between cells and the difference in their tuning properties results in more tightly clustered cell ensembles when compared to a non-columnar architecture. What this means is that since cells preferentially connect to others with similar tuning [17] and cells with similar tuning are physically close to each other in a columnar cortex, these cells will find more appropriate targets and thus form more densely connected ensembles compared to the case in a non-columnar cortex. The model shows that columnar architecture results in a cortical network that is more resistant to noise, both general and input-specific, than a cortical network without columns.

## Methods

We constructed a simple idealized model focused on demonstrating the differences between cortical networks with and without columnar architecture, rather than providing absolute quantitative results regarding cortical circuitry per se. The essential feature of columnar architecture is that neighboring cells tend to have similar parameter tuning, which can be studied within a single layer. The vertical “columnar” property arises from the fact that maps in superficial and deep layers are in register [13]. We used data from cats where it exists, otherwise data from other mammals was used for basic properties that would not be expected to differ, for example the inverse relationship between cell connection probability and physical distance.

### Columnar architecture

We first consider consequences that arise solely from the input to the cortical field. At least half a dozen parameters (features) show clustering, thus are mapped across the surface of cat primary visual cortex [2]. The strongest map is that of orientation; that is, the preferred orientation of neighboring cells shows the least variance of all features. There are strong maps for some other features such as spatial frequency and ocular dominance, and these maps tend to be orthogonal to each other [4], [18], [19], which is thought to provide complete feature coverage at every point in the visual field [6]. Some features have also been identified that show weak or no mapping, such as spatial phase [2], [20].

We modeled these data as two orthogonally-mapped circular variables representing the value of the preferred stimulus for each cell. Every cell was assigned a preferred orientation (*orient*) according to its position on the horizontal axis (0 to 180 degrees) with gaussian noise added:

$$orient=m+SD\sqrt{-2ln(x)}cos(2\pi y)$$ (1)

where *m* is the distance along the axis, *SD* is the standard deviation and *x* and *y* are uniform random variables between 0 and 1.

**Fig 1:**
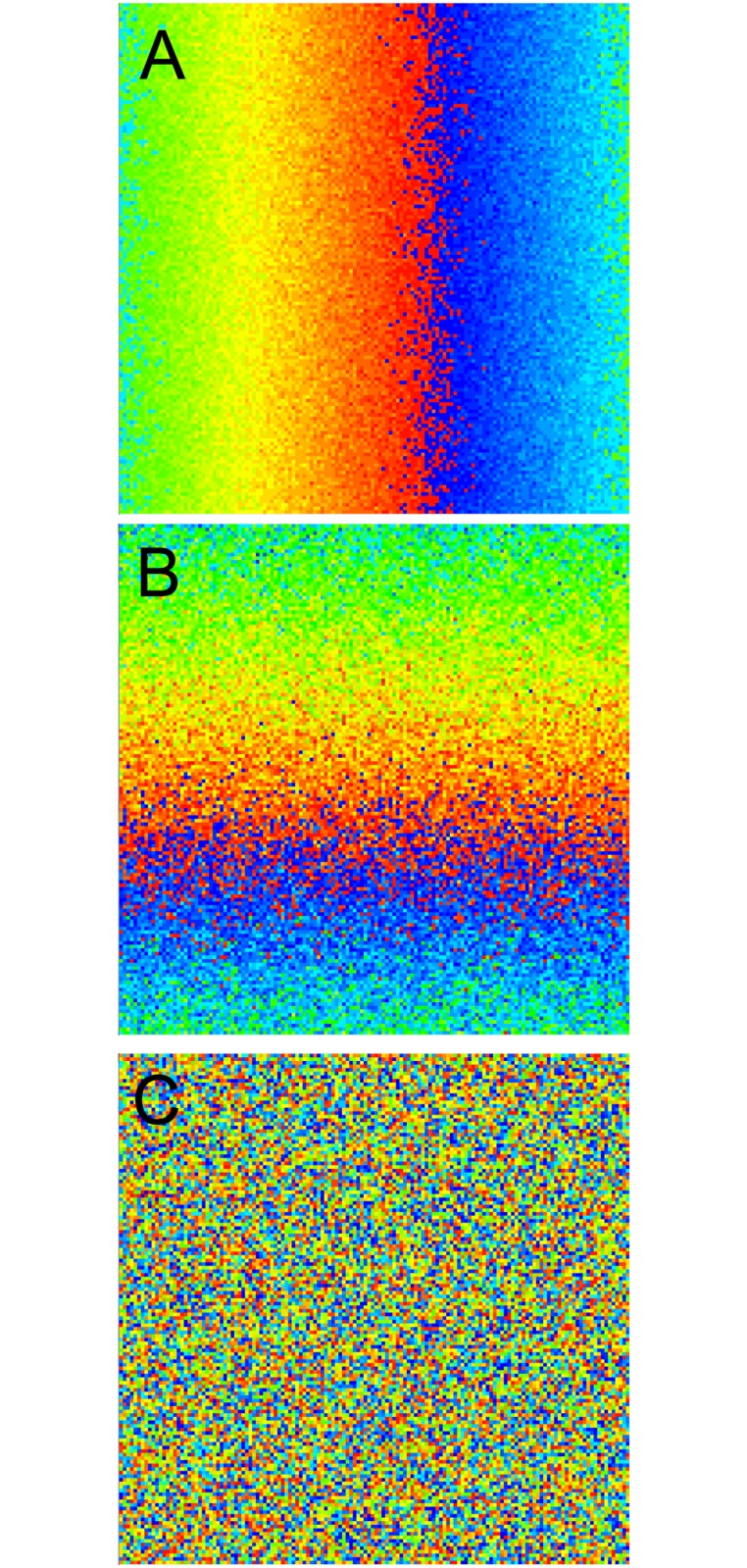
Model columnar architecture. An optimal (preferred) value for each of four stimulus parameters was assigned to each cell (20164 total). A: Orientation preference was distributed horizontally with the least noise in the distribution. B: The second parameter was distributed vertically with a more noisy distribution. C: The third and forth parameters were distributed randomly to represent stimulus features that are poorly or not mapped. Each square is 1mm per side.

**Fig 2:**
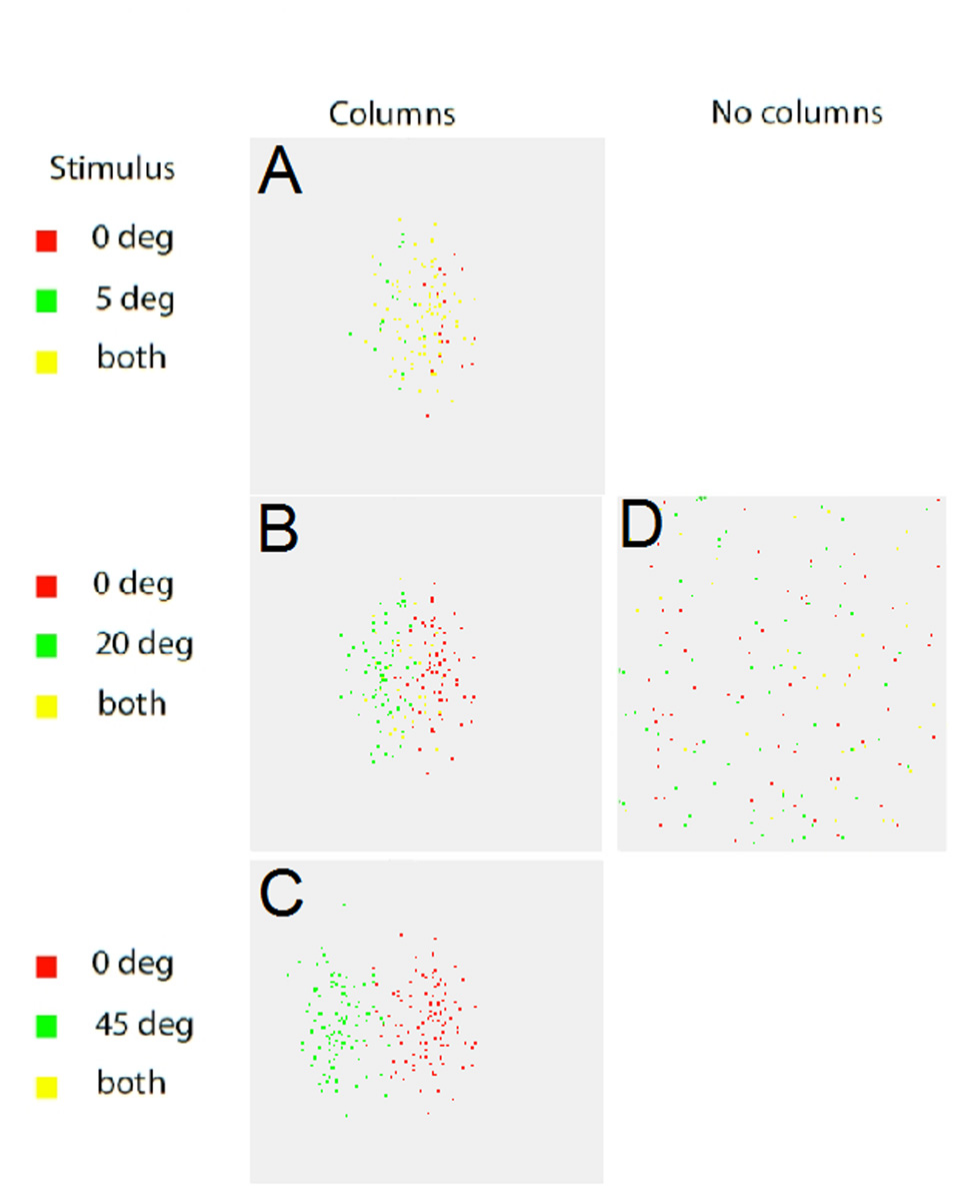
Overlap in feedforward tuning. In red are the 100 cells best tuned to the reference orientation (0 degrees), in green the 100 cells best tuned to the test orientation. Yellow shows cells common to both populations. Feedforward inputs only (no intracortical connections). A: For a stimulus difference of 5 degrees the two populations overlap by 83%. B: This overlap reduces to 32% for an orientation difference of 20 degrees. C: Although there is still some overlap in the physical positions of the cells, the two populations best tuned to stimuli 45 degrees apart do not share any common cells. D: The non-columnar network shows the same degree of overlap (35%) for a stimulus difference of 20 degrees but the best tuned cells are scattered randomly rather than clustered.

The standard deviation was smallest (7 degrees) for orientation, the most strongly mapped parameter. The second parameter was mapped in the same way along the vertical axis with a larger SD (10% of parameter range). A third and a fourth randomly distributed variable were added to represent weakly- or non-mapped features (Fig. 1). To implement a non-columnar model all four variables were randomly distributed (no mapping). Thus, each cell can be characterized by four parameters representing the preferred values of the four features, forming a four-dimensional feature space. The Euclidean distance between the points corresponding to two cells within this space gives a measure of the difference in their tuning. Each stimulus can be considered as a point in this space, allowing each cell to be assigned an input based on its tuning:

$$g_{in}=\frac{1}{\sqrt{2\pi \sigma^{2}}}e^{-\frac{td^{2}}{2\sigma^{2}}}C$$ (2)

where *g*_*in*_ is a constant conductance input applied to the distal dendrites of each pyramidal cell, σ^2^ is the variance (0.1) set to give a realistic value for the width of the orientation tuning curves (approximately 30 degrees HWHH), *C* = 15 and *td* is the tuning distance:

$$td=\sqrt{\sum_{i=1}^{4}{\left(X_{is}-X_{ic}\right)}^{2}}$$ (3)

where *i* indexes the 4 parameters shown in Fig. 1 and *s* and *c* refer to the stimulus and cell respectively. X_1c_ is “orient” from Equation 1. All parameters were normalized to the same value before computing *td*. Assignment of four parameters to each cell according to its spatial location implements a columnar architecture with those four parameters specifying the optimal stimulus for each cell. Conversely, we can take any stimulus and determine the N cells best tuned to this stimulus (shortest Euclidean distance of the cell's parameters to the stimulus parameters). Fig. 2 shows the 100 best-tuned cells, in terms of purely feedforward input (no dynamics or connections), for a series of pairs of inputs of increasing orientation difference. For two stimuli differing by 5 degrees there is substantial overlap (Fig. 2A); 83% of the best-tuned cells are shared by the two populations. Two representations composed of so many of the same cells will likely be hard to discriminate. The orientation discrimination threshold of the cat is about 5 degrees [21], which may be because representations of stimuli closer in orientation than this share too many cells. When the difference increases to 20 degrees (Fig. 2B) the overlap is reduced to 32%. At a stimulus difference of 45 degrees there is no overlap even though the two populations are still somewhat physically overlapped (Fig. 2C). Fig. 2D shows the result for a 20 degree stimulus difference for the non-columnar network, with approximately the same overlap (35%) as the columnar case but with the 100 cells distributed randomly across the space instead of grouped together [22].

### Connectivity

**Fig 3:**
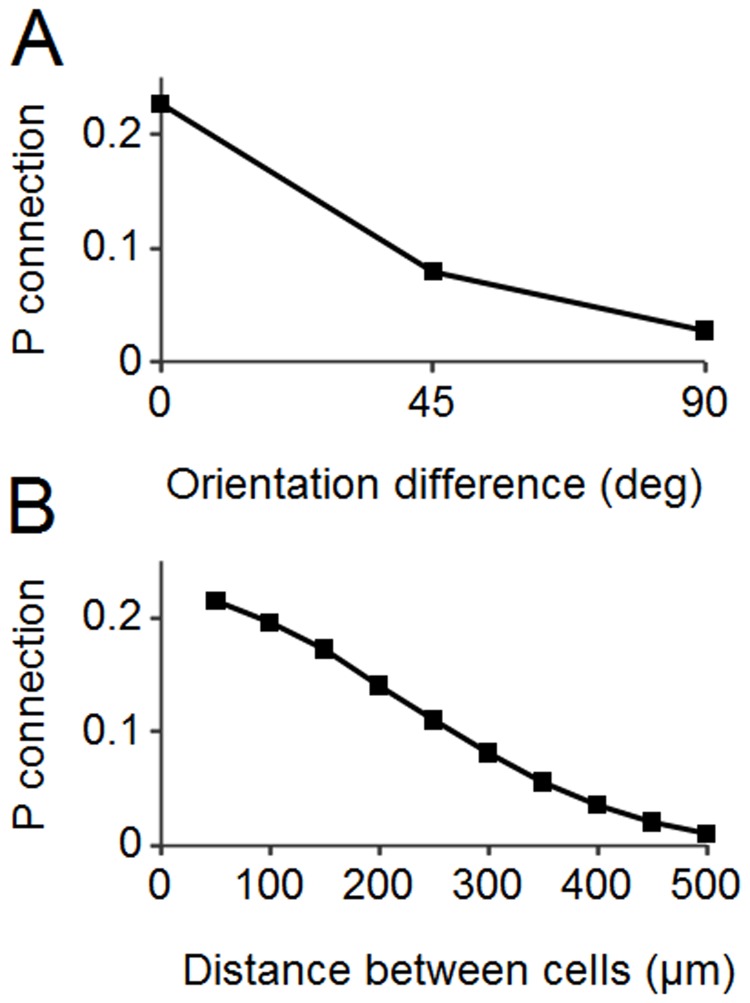
Connection probabilities of pyramidal cells in model. A: The dependence of the connection probability of two cells in the non-columnar network on the difference in their preferred orientations. B: The dependence of the connection probability on the physical distance between the cells. Both relationships are approximately inversely linear.

Next we consider the impact of intracortical connectivity. We first assumed that the probability of connection (Fig. 3) was equal to the product of the inverse of the physical distance between the cells [15], [16] and the inverse of the difference in their tuning [17]. The Ko et al. study was performed in rodents and although a direct demonstration of this relationship has not yet been done in columnar animals, it has long been suspected [23]. The Holmgren and Oswald studies were also done in rodents and we assume that this relationship, which is due to geometrical constraints, obtains in other mammals. Physiological and anatomical data [24], [25] indicate that direct pyramidal-pyramidal connections within a column extend to about 500 μm. Thus, pyramidal cells were connected with a probability given by:

$$P(connection)=(1-\frac{pd}{pdMax})(1-\frac{td}{tdMax})$$ (4)

where *pd* is the physical distance between the two cells (minimum 7 μm), *pdMax* = 600, *tdMax* = 1.1 and *td*, the tuning distance between the cells, ranged from 0 to *tdMax* [17], [24], [25]. *tdMax* is a determinant of the slope in Fig. 3A. We found that a value of 1.1 (from a range 0 to 2) gave the best fit to the experimental data (eg. Fig. S6D of ref 17). Connections were wrapped around boundaries (effectively forming a 3-D toroid) to avoid edge artifacts.

**Fig 4:**
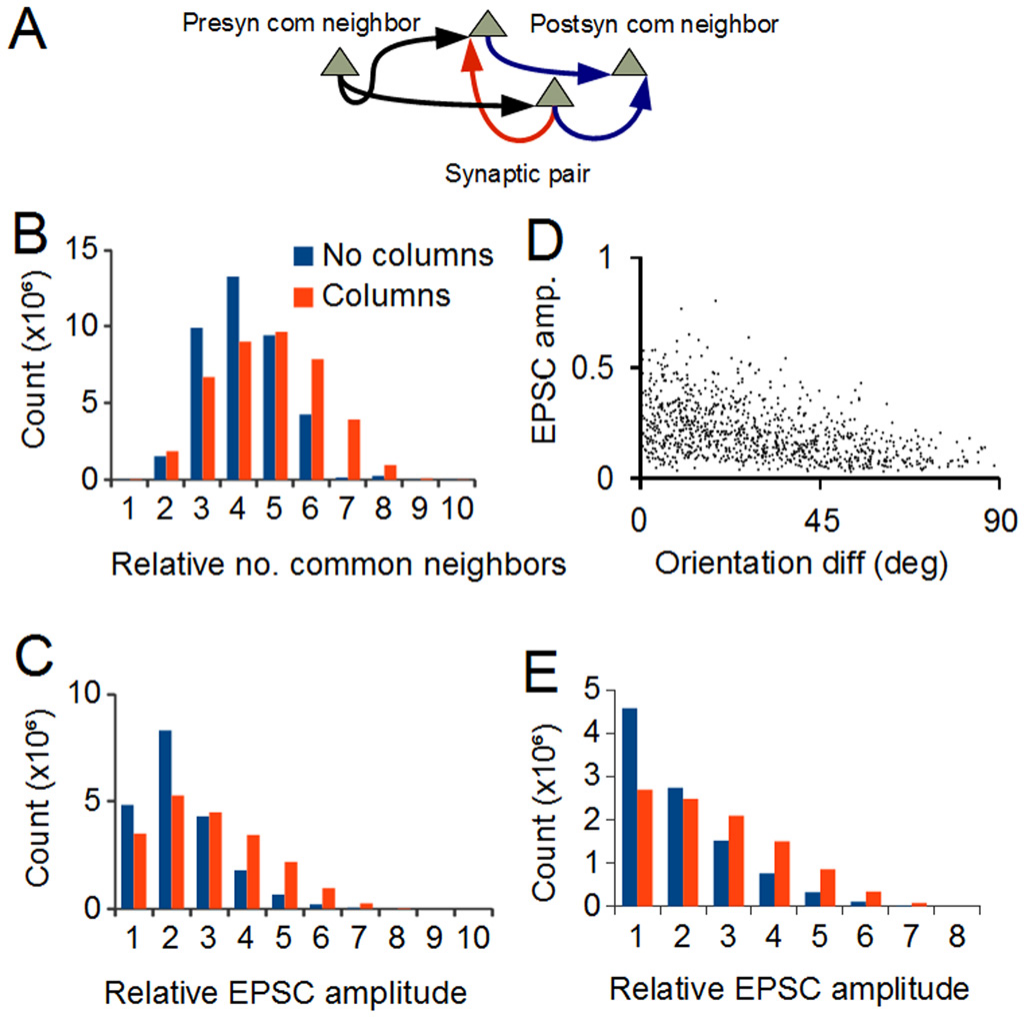
Calculation of EPSC amplitudes. A: For a synaptically coupled pair of cells (red arrow), presynaptic common neighbors are those cells that project to both members of the pair (black arrows). Postsynaptic common neighbors are those cells that are projected to by both members (blue arrows). B: Number of common neighbors (pre + post) in the non-columnar (red) and columnar (blue) networks. The columnar network has relatively fewer pairs with low numbers of common neighbors and more pairs with high numbers. This is a metric of the clustering of cells in the two networks. C: The strength of the synaptic connection between two cells is the product of the number of pre and postsynaptic common neighbors. The columnar network has more strong synapses than the non-columnar, reflecting its greater degree of clustering. D: Dependence of synaptic strengths for all 1000 synapses made by a cell in the non-columnar network on the orientation difference between that cell and its targets. Values normalized to population maximum. There is a tendency towards an inverse relationship although there is significant noise in the distribution, as in the physiological data (Ko et al 2011). The stronger synapses are those between cells forming part of a tightly clustered ensemble. E: Distribution of synaptic strengths in a model where EPSC amplitude is simple Hebbian (given by the similarity in tuning of the two cells). The similarity to C shows that the detailed formulation of EPSC amplitude is not critical in producing our results, what is crucial is the dependence of EPSC amplitude on feature similarity.

Functional connectivity depends not only on the anatomical connections but also on the strengths of the synapses. The amplitude of a synaptic connection (given by synaptic conductance in the model) between two layer 2/3 pyramidal cells is not completely determined by the similarity of their tuning or the correlation in their firing rates ([17], their Fig. S4) contrary to the expectations of a simple Hebbian model, in which spike timing correlation would be the sole determining factor. Recent experimental work shows that the amplitudes of synaptic connections between cortical pyramidal cells are proportional to the tightness of clustering of the local population within which the connections are embedded [26] (connections within layer 5). This tightness, quantified as the number of common neighbors (the number of cells projecting to or from both cells of a synaptically-connected pair, Fig. 4A), has only been experimentally determined up to a number of 2 or 3 neighbors, producing a probabilistic relationship ([26], their Fig. S5b). In the model, however, all the common neighbors of each synaptic pair can be counted exactly (Fig. 4B), allowing a precise calculation of EPSC amplitudes based on this metric. The strictly deterministic relationship implied here may not be exact in reality, but is used here as an approximation. EPSC amplitude for a connection was therefore calculated using the product of the number of cells projecting to both the members of the synaptically-connected pair and the number of cells projected to by both members:

CN=nPre nPost (5)

where *nPre* is the number of presynaptic common neighbors (normalized to the maximum value) and *nPost* is the normalized number of postsynaptic common neighbors. Multiplication was used in preference to simple addition to provide a form of logical AND gating; thus large amplitude EPSCs would only result from connections where both the number of presynaptic and postsynaptic common neighbors was high.

Perin et al. also show that there is a (rapidly saturating) dependence of EPSP amplitude on the total number of connections between all the members of a cluster (their Fig. 5A). Thus, we need to calculate a metric of the degree of clustering of the immediate network in which the synapse in question is embedded. We can take advantage of the fact that counting connections among the synaptic targets of each cell has already been done implicitly when calculating numbers of common neighbors above.

**Fig 5:**
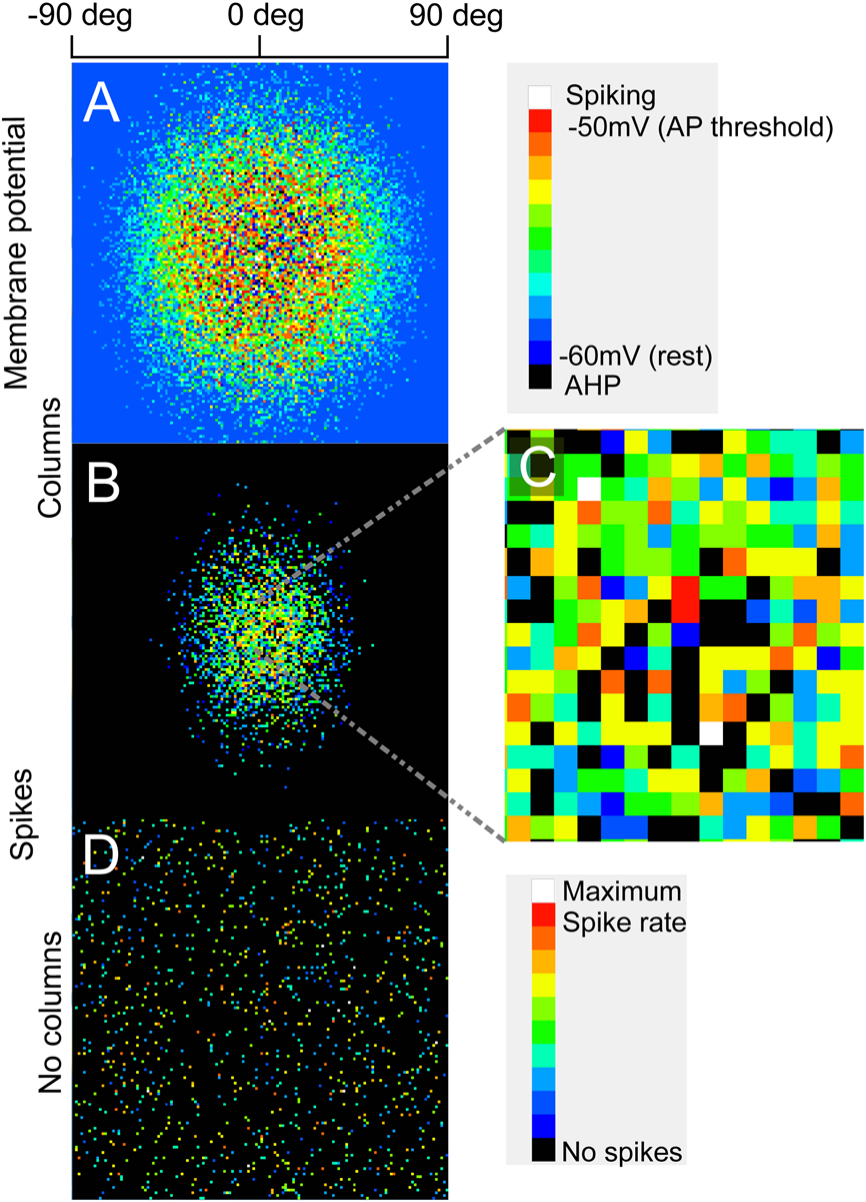
Network response to input. A: Membrane potential of all cells in columnar network after 200ms of stimulation at an orientation of 0 degrees. Scale shows preferred orientation. AP is action potential, AHP is afterhyperpolarization. Subthreshold depolarization is extensive, as far as cells preferring orthogonally oriented stimuli. B: The same simulation as A showing spike rate instead of membrane potential. Spiking is restricted to cells with preferred orientations within about 45 degrees of the stimulus. C: An expansion of the center region of B shows that the spiking in the center of the column is heterogeneous, with cells firing maximally adjacent to cells not firing at all. D: Spike rates to the same stimulus for the non-columnar network. Spiking cells are distributed randomly across the space. Scale bar at top shows mean preferred orientation of the cells.

Therefore, we define *nPreMean* as the mean number of presynaptic common neighbors for a cell and all of its targets and *nPostMean* as the mean number of postsynaptic common neighbors. Then to produce a metric of cluster connectivity for any cell and a particular target we calculate *ccPre* and *ccPost* for the target cell, taking the log to account for the strong saturation:

ccPre= ln(nPreMean) +CccPost= ln(nPostMean) +C (6)

where *C* = 3, with cluster connectivity (*CC*) given by:

$$CC=\frac{\left(ccPre+ccPost\right)}{2}$$ (7)

Because of the logarithmic nonlinearity the effect of this term is small and EPSC amplitude is dominated by Equation 5. Given B, the base excitatory postsynaptic conductance given in Table 1, EPSC amplitude is:

$$EPSC\;amplitude=CN^{}CC^{}B$$ (8)

For simplicity, and considering the small contribution of Equation 7, we treat CN and CC as independent although they may be interrelated.

**Table 1:** Model Parameters.

Resting potential	-60 mV
Action potential threshold	-50 mV
Excitatory synaptic conductance reversal potential	0 mV
Inhibitory synaptic conductance reversal potential	-60 mV
Excitatory synaptic delay	1.3 ms
Inhibitory synaptic delay	2.5 ms
EPSC duration	0.5 ms
IPSC duration	2 ms
Base excitatory postsynaptic conductance	0.5 nS
Inhibitory postsynaptic conductance	0.01 nS *
Membrane time constant	20 ms
Proximal input resistance	100 MΩ
Medial input resistance	250 MΩ
Distal input resistance	300 MΩ
Axial resistance	4 MΩ

* Each pyramidal cell spike effectively produces an IPSP in every pyramidal cell; see Methods.

Parameters used in biophysical model, based on [70] review.

The resultant distributions are shown in Fig. 4C. Columnar architecture shifts the distribution to the right, increasing the number of synapses with large amplitude EPSCs, producing stronger cell ensembles in the columnar network. The amplitudes of all the synapses made by a cell of the noncolumnar network are shown in Fig. 4D. The model shows the same mean tendency of inverse proportionality to orientation difference as the experimental data [17] with considerable noise.

While not strictly Hebbian at the single cell level (as experimental data indicates [17], [27], [28]) this scheme may be Hebbian at the population level, i.e. synaptic connections increase in strength based on coincident activity of the cluster in which they are embedded.

In order to demonstrate that these results are not dependent on the particular formulation of EPSC amplitudes used in our model, Fig. 4E shows EPSC amplitude distributions for simulations using a simple Hebbian rule, i.e. EPSC amplitude directly equal to the similarity in tuning parameters (orientation, etc.). The fact that the same relationship holds, i.e. the columnar network EPSC distribution is still shifted to the right relative to the non-columnar case, indicates that the details of the EPSC amplitude calculation we use are not critical; it is only necessary to have some dependency of EPSC amplitude on parameter similarity. We are not making the claim that we have captured the exact equations that determine EPSC amplitude, rather we claim that the details of EPSC amplitude determination are not relevant to the main result of our study, which is essential a geometrical one given at the end of the INTRODUCTION and at the beginning of the RESULTS. The formulation we use provides EPSC amplitude distributions and variances matching the experimental data and is based on a clustering metric shown to be correlated with EPSC amplitude.

### Biophysical simulations

The number of pyramidal neurons under a square millimeter of cortical surface in layer 2/3 of cat primary visual cortex is just over 20,000 [29]. Our model consisted of 20164 pyramidal neurons. Rather than using the integrate and fire single point neuron common in large network models, each pyramidal cell was simulated as a three-point neuron, with the three equipotential points corresponding to the three somatodendritic regions of a pyramidal cell: (1) the soma, initial axonal and dendritic trunks, (2) proximal (aspiny) dendrites and (3) distal (spiny, terminal) dendrites. This allowed placement of excitatory synapses on distal dendrites and inhibitory synapses on the soma and proximal dendrites (each inhibitory connection effectively making two synapses on each pyramidal cell, each with conductance given in Table 1), which results in shunting inhibition and dendritic saturation [30]. Thus, accuracy was increased compared to a single point neuron without overly adding to the simulation time and complexity (e.g. each point was characterized by an input resistance, rather than a full compartmental simulation). Model parameters are shown in Table 1.

We used standard voltage-current equations [31], [32]:

At each point the membrane current *I*_*m*_ is given by

$${\text{I}}_{\text{m}}=g(V_{rev}-V_{m})$$ (9)

where *g* is the synaptic conductance calculated in Equation 8 for excitation and the inhibitory synaptic conductance given in Table 1 for inhibition, *V*_*rev*_ is the excitatory/inhibitory reversal potential given in Table 1 and *V*_*m*_ is the membrane potential.

At each point the axial current I_a_ is given by

$$I_{a}=\frac{\left(V_{1}-V_{m}\right)}{R_{a}}$$ (10)

where R_a_ is the axial resistance given in Table 1 and V_1_ is the membrane potential of the adjoining point (two adjoining points when considering the medial point).

The membrane potential at each point evolves according to

$$\tau_{m}\frac{dV_{m}}{dt}=-V_{m}+R_{in}({\text{I}}_{\text{m}}+I_{a})$$ (11)

where τ_m_ is the membrane time constant and R_in_ is the input resistance, both given in Table 1.

Inhibitory neurons were not explicitly modeled; rather inhibition was simulated as the sum of excitatory activity [33], [34], each action potential in a pyramidal cell producing an EPSC in its targets after a delay and an IPSC in all pyramidal cells after a longer delay (see Table 1). This reflects the dense nature of local intralaminar cortical inhibition [15], [17], [29]. The effect of varying the strength of inhibition is described in RESULTS. We found that the strength of excitation was narrowly constrained: if the value of the base excitatory postsynaptic conductance (Table 1) was halved then recurrent excitatory feedback had very little effect (not shown). However, if excitation was doubled then the network locked up with all cells firing strongly (not shown). If this was compensated with increased inhibition then the network displayed unphysiological global all-or-none oscillations.

Synaptic kinetics were simplified to a rectangular pulse conductance change (duration 1ms) following the presynaptic spike (after a delay) because the model included over 2 x 10^7^ synapses that were the rate-limiting factor in the simulations. The voltage-current equations were integrated using the 4^th^-order Runga Kutta method with a time step of 10 microseconds which provided a high degree of accuracy and stability.

### Measuring relative performance

In order to determine the functional difference between the columnar and noncolumnar networks, we considered two performance metrics, the relative discriminability of stimuli of different orientations and the noise robustness of the two networks. To measure performance we used the Pearson correlation coefficient:

$$r=\frac{\sum_{i=1}^{n}\left(X_{i}-\bar{X}\right)\left(Y_{i}-\bar{Y}\right)}{\sqrt{\sum_{i=1}^{n}{\left(X_{i}-\bar{X}\right)}^{2}\sum_{i=1}^{n}{\left(Y_{i}-\bar{Y}\right)}^{2}}}$$ (12)

where X_*i*_ is the number of spikes fired by cell i in population *X*, *Y*_*i*_ is the number of spikes fired by cell i in population *Y* and$\overset{-}{X}$, $\overset{-}{Y}$ are the averages. This measure is preferred to determine the similarity of the spiking response of two neural populations since it is not sensitive to the absolute firing rate of the populations and provides performance equivalent to more sophisticated analyses [35]. In discriminability trials we took the Pearson correlation coefficient (similarity) between the spiking response of the entire network to a reference orientation stimulus and the response to a stimulus of a different orientation. We assume discriminability will be a monotonically inverse function of response similarity, depending on trial-to-trial noise. In noise robustness trials we measured the similarity between two responses to the same orientation, one with noise and one without. Correlated noise cannot be removed by the simple cortical circuitry described here [36] and is likely very low in awake animals [37], [38], thus we considered the effect of uncorrelated noise.

The noise robustness exhibited by our network is that demonstrated by connectionist pattern associators, essentially the property of stabilizing a particular ensemble of neurons against noise (input variability). The same property also bestows fault tolerance and pattern completion properties as well as fast convergence on the correct pattern [39].

We are focused on determining whether columnar cortex is more robust to noise than non-columnar cortex rather than making quantitative claims about the degree of noise robustness in cortex in general. Therefore we used large amplitude noise to produce a significant effect against which noise recovery could be measured. Noise was of two types: input specific, in which the amplitude of *g*_*in*_ from Equation 2 was randomly increased or decreased by 33%, and nonspecific noise, in which every pyramidal cell had a noise conductance of 5nS, added to *g*_*in*_, that turned on with a probability of 0.0005 every millisecond and was set to zero with p = 0.001 every millisecond, producing a steady state in which 33% of randomly chosen cells had this conductance active. Both types represent intrinsic noise, eg. variability in the firing of retinal, LGN, layer 4 and other cortical inputs. In the results presented here all noise trials include both types of noise.

### Synaptic failures

Medium and large amplitude synapses between neocortical pyramidal cells are very reliable, while weak synapses show failures of transmission up to 100% for the weakest [16], [40]. The model incorporated this phenomenon by multiplying the amplitudes of synapses by a piece-wise linear approximation to the data (Fig. 12A, based on Fig. 6C of [16] and Fig. 3E of [40]), with medium and strong synapses unmodified and weak synapses reduced in strength proportional to their failure rate. In order to discover the functional effect of synaptic failures, we also performed simulations where this correction was not applied, i.e. the line in Fig. 12A is completely flat at zero failures. Since this had no effect on noise robustness we ran a control where, rather than reducing EPSC amplitudes of the weakest synapses (0–20^th^ amplitude percentile) as in Fig. 12A, we reduced the amplitudes of medium strength synapses (20^th^—40^th^ percentile). We did this using the same piece-wise linear function except with the sloped part only for the synapses with normalized amplitude 0.2–0.4, zero elsewhere.

**Fig 6:**
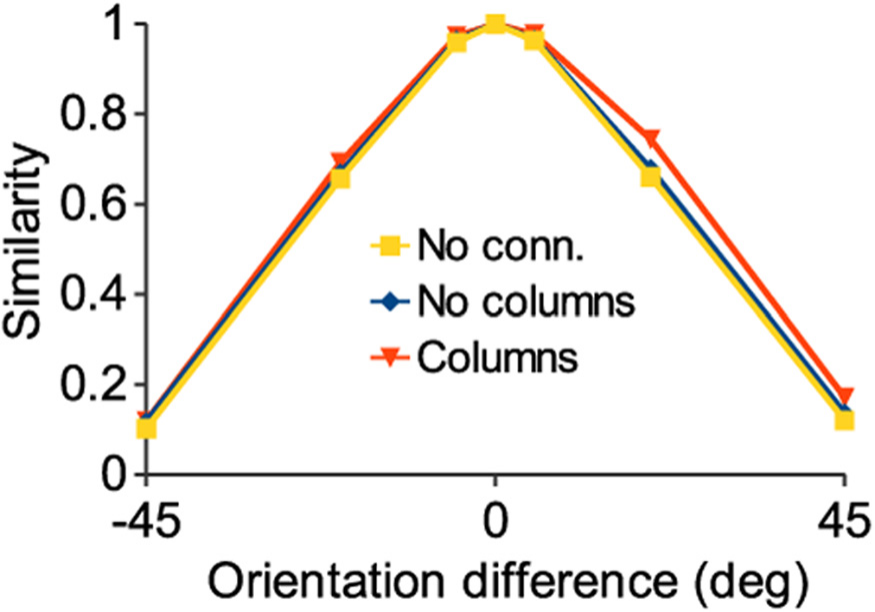
Recurrent connections do not impair discrimination. Similarity of network population response at different test orientations relative to the response at an orientation of 0 degrees. We take the inverse of this measure as a metric of the discrimination ability of the network. The addition of intracortical connections does not change the similarity of responses significantly, implying that the discrimination power of the network is unchanged.

## Results

The model was constructed with the following assumptions, based on experimental data: Columnar architecture can be modeled with noisy one-dimensional linear maps of preferred stimulus parameters positioned orthogonally to each other (Fig. 1A,B; Fig. 2A,B,C). Non-columnar networks have preferred parameters that are randomly distributed in space (Fig. 1C; Fig. 2D). Connection probability between cells is a function of preferred stimulus similarity and physical proximity (Fig. 3). Synaptic strength is a (noisy) function of preferred stimulus similarity (Fig. 4).

As a result of these constraints, cells that prefer similar stimuli are more likely to be connected and with stronger synapses. In a columnar network these cells are physically close to each other whereas in a non-columnar network they are at random distances (within approximately 1mm). Thus, a columnar network will consist of ensembles of cells that are more strongly connected (clustered) than a non-columnar network. We hypothesized that this greater degree of clustering would be manifested as improved network performance, specifically when considering robustness to noise.

### Network response to input

To test this, the columnar and non-columnar networks were tested with input of a specific orientation, simulated for a period of 200ms. Population responses to different orientations were compared to illustrate discrimination power and responses to the same orientation with and without noise were compared to show the noise robustness of the two networks (see Methods). We first briefly describe the general response characteristics of the network to input:

Fig. 5A shows the membrane potential of all pyramidal cells in the columnar network at the end of a 200 ms stimulation period with parameter values of the stimulus all set to half maximum, which was designated as 0 degrees orientation for the first parameter. Subthreshold depolarization is extensive, even extending to cells with an orthogonal orientation preference from the stimulus. Fig. 5B shows that the spiking response for the same cells is much more restricted [41]; no cells with a preferred orientation greater than about 45 degrees different from the stimulus fire any spikes. Fig. 5C, an expansion of the central area of Fig. 5B, shows the significant heterogeneity in spike rates between cells, even at the center of a “column” [20], [42]. Cells spiking at maximum rates are adjacent to cells firing no spikes to the same stimulus because although the ordered mapping shown in Fig. 1 holds over millimeters, at a smaller scale the most active members of a cell ensemble responding to any particular stimulus may be randomly distributed over hundreds of microns. The heterogeneity in the model is present with just four stimulus dimensions and without any external (extracolumnar) inputs. It is due to a combination of the variance in the parameters of the stimulus dimensions (Fig. 1) and the probabilistic nature of the connectivity. Fig. 5D shows the spiking response of the noncolumnar network to the same stimulus, displaying a “salt and pepper” distribution of spiking cells over the whole square millimeter, characteristic of the rodent [22].

### Discrimination

We determined whether columnar architecture affects the ability of the network to discriminate stimuli, taking response similarity as the metric (see Methods). The response similarities of the columnar and non-columnar networks, as well as a network with no synaptic connections, are shown in Fig. 6. As expected, the similarity of the spiking responses decreases as the orientation difference between the two stimuli increases. Perhaps less expected, the performance of the connected networks is not significantly worse than the network without connections. This is despite the fact that, because of strong excitatory feedback connections, the columnar network fires around twice as many spikes as the unconnected network to the same stimulus. The addition of these excitatory connections between cells might be expected to result in worse discrimination of similar inputs due to introduced correlations [43].

**Fig 7:**
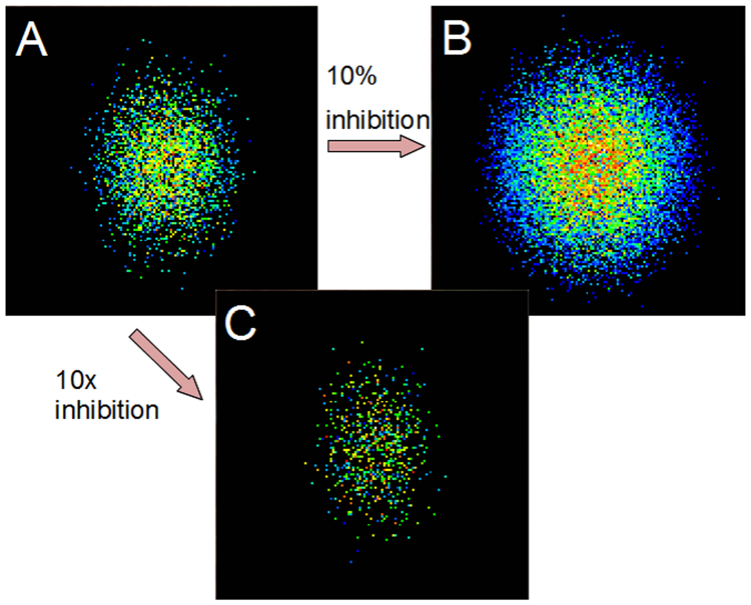
Effect of changing strength of inhibition. A: This is the same plot as in Fig. 5B, showing a population response with default inhibition. B: When inhibitory strength is decreased to 10% of default the response expands as many more cells are recruited. C: Increasing inhibition by 10x contracts the response, allowing only the best tuned cells to fire.

**Fig 8:**
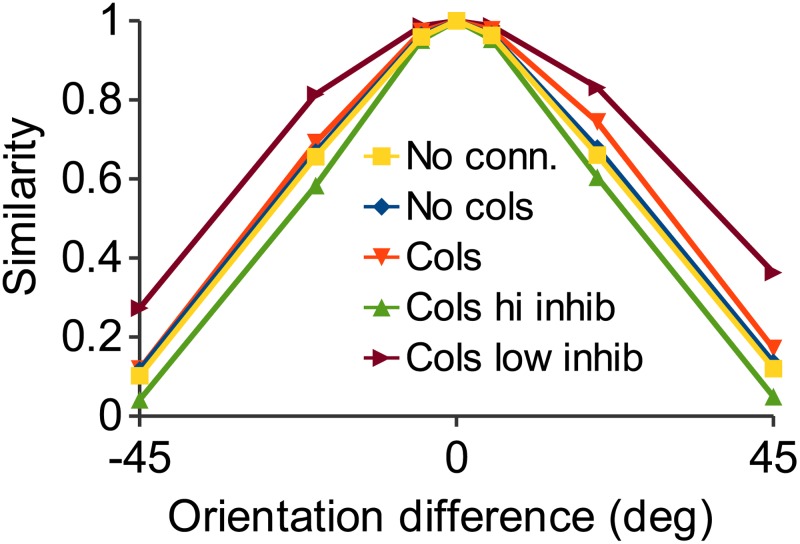
Increasing inhibition improves discrimination. Response similarity as in Fig. 6. The plot for decreased inhibition is from the data of Fig. 7B. Response similarity is increased implying worse orientation discrimination. Increased inhibition, from the data of Fig. 7C, shows response similarity is decreased, implying that discrimination is enhanced by increasing inhibition. Both test plots are from the columnar network in both this figure and Fig. 10.

We hypothesized that the reason that response similarity was not increased by intracortical connections was because of the recruitment of inhibitory feedback. While excitatory interconnections increase the firing of the cells best tuned to the stimulus due to the tuning-dependent connectivity (see Methods), inhibitory feedback suppresses the firing of cells less well tuned to the stimulus, decorrelating the output [44], [45]. To test this, we modulated the strength of inhibition in the model. Fig. 7A shows the same response as Fig. 5B, with default inhibition. Fig. 7B shows that decreasing inhibition by an order of magnitude causes a spread of the firing response to non-preferred orientations [46], [47]. Completely eliminating inhibition causes the activity to spread to every pyramidal cell in a paroxysmal response (not shown). Increasing inhibition by an order of magnitude (Fig. 7C) has the opposite result: the size of the spiking population contracts as many more cells are inhibited. Fig. 8 shows the same response similarity functions as Fig. 6 with the addition of the results for the increased and decreased inhibition cases. Decreasing inhibition increases response similarity while increasing inhibition has the opposite effect. Thus, it might be thought that maximal inhibition would be optimal in order to produce the greatest discriminability of stimuli. However, this leads to suboptimal performance under noisy conditions, as shown below.

### Robustness to noise

**Fig 9:**
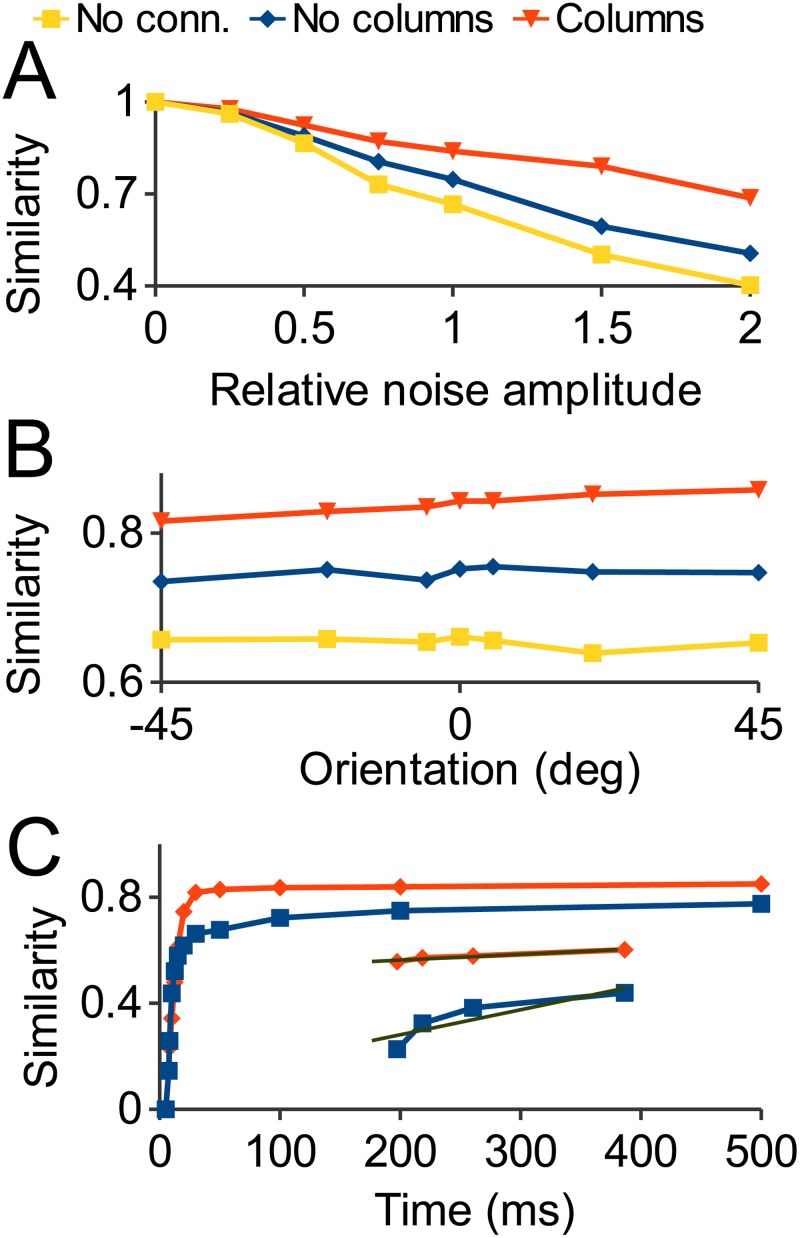
Noise robustness of recurrent network. A: Similarity of network population response with noise added relative to the response without noise (all stimuli 0 degrees orientation). A value of 1 means total noise recovery. Adding connections improves noise robustness, with the columnar network performing significantly better. B: Similarity of response to stimuli at different orientations with noise added compared to responses to the same stimuli without noise. Columnar network performs best at all orientations. C: Similarity of non- (blue) and columnar (red) network response with noise compared to no noise tested at different time intervals. The columnar network converges on the asymptotic performance level faster than the non-columnar network (inset shows blow up of last 4 data points with regression lines).

Next we tested to see if columnar architecture has an effect on the disruption of neural representations by noise. Fig. 9A shows the noise sensitivity of the columnar and noncolumnar networks; the performance of the unconnected network is included as a baseline to show the effects of increasing levels of noise. Addition of noise (see Methods) to the network reduced the similarity of the response relative to the no noise case significantly (yellow line). Performance was improved somewhat when the non-columnar connections were added (blue), suggesting that the excitatory connections functioned to recover the ensemble activated in the no noise case.

The columnar network showed significantly further improved performance (red line) compared to the non-columnar case. The stronger ensembles with stronger synapses (Fig. 4) between the elements were better able to restore a noisy input. The activated ensemble primarily consists of the cells best tuned to the stimulus: All of the 100 cells best tuned to the 0 degree stimulus shown in Fig. 2A have firing rates in the top 30^th^ percentile in the no-noise case and in the top 44^th^ percentile in the noise case shown in Fig. 9.

Fig. 9B shows results for stimuli over a range of orientations at the relative noise amplitude of 1. The columnar network outperformed the noncolumnar at every orientation. The structured pyramidal-pyramidal cell connections in the model also reduced the time taken to converge on a stable representation: Fig. 9C shows network performance over time. The columnar network reached 95% of the asymptotic level noise recovery by 30 ms, while the non-columnar network took almost 150ms to reach same level of performance.

### Importance of network functional structure

**Fig 10:**
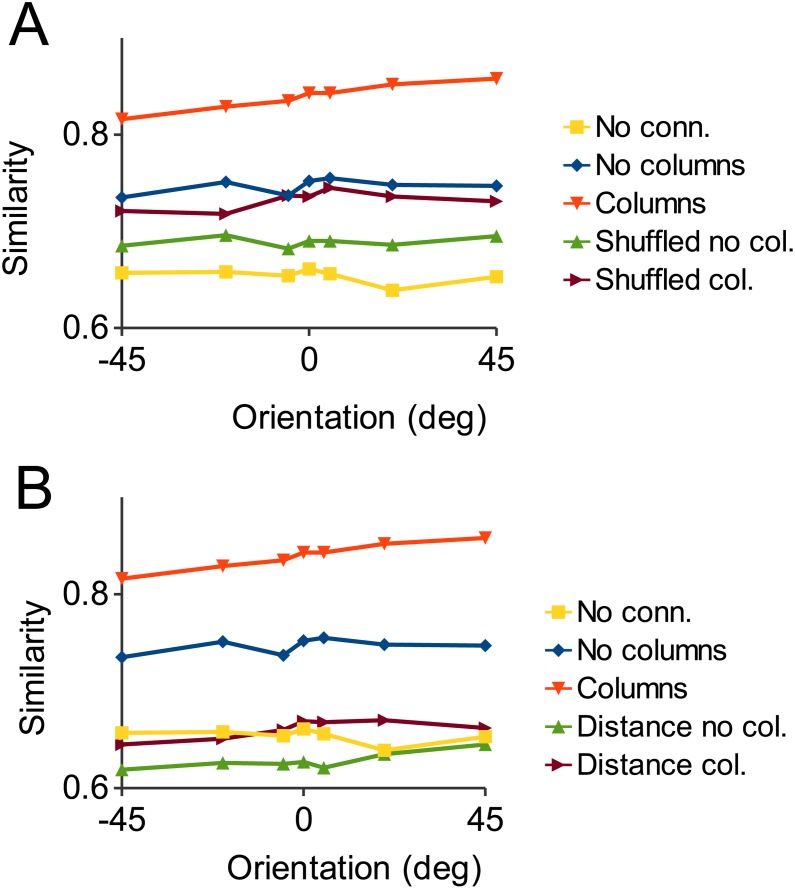
Noise robustness is dependent on functional structure of cell ensembles. A: Shuffled results overlaid on the graph of Fig. 9B. These are the results of simulations in which the amplitudes of synaptic connections have been randomly shuffled. This preserves total number and strength of connections but significantly degrades the noise robustness of the networks. B: As in A, except the distance plots show results for networks in which probability of connection is only dependent on inter-neuronal distance, not feature preference. In this case noise robustness is no better than the unconnected network.

In order to test the importance of the fine synaptic structure of the network we performed simulations in which the synaptic strengths of pyramidal cell connections are shuffled (i.e. the strengths of two randomly chosen synapses were swapped, repeated 4x10^7^ times, preserving the total number and strength of connections). These simulations show that performance of the shuffled network is severely degraded relative to the control case (Fig. 10A). Similarly, if the probability of connection between pyramidal cells is only dependent on the distance between the cells and not on their feature preference, while preserving total number of connections, then performance is no better than that of an unconnected network (Fig. 10B). These results show that it is the specific functional connectivity within an ensemble of similarly tuned cells that produces strong robustness to noise: It is necessary that the most densely interconnected (clustered) cells forming an ensemble, close neighbors in parameter (feature) space, are connected with the strongest synapses.

### Noise robustness with varying inhibition

**Fig 11:**
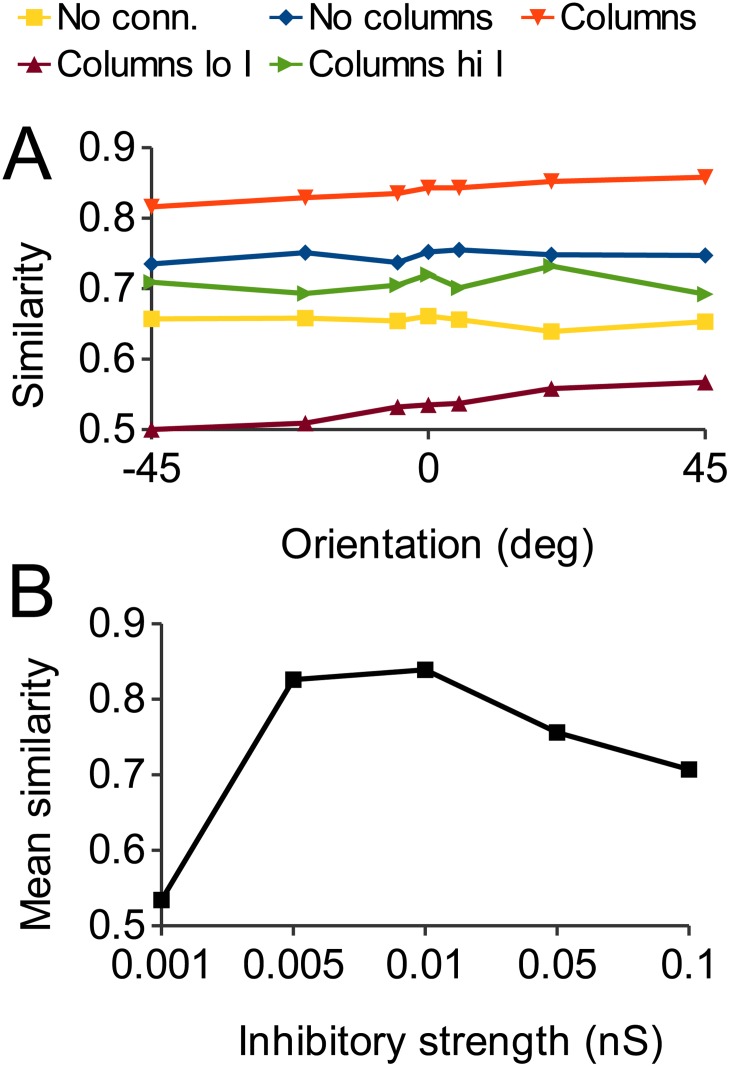
Effect of changing inhibition on noise robustness. A: Response similarity as in Fig. 9B. The low inhibition plot is the result for decreased inhibition shown in Fig. 7B. Response similarity is much lower, indicating severely impaired noise robustness. The high inhibition plot is the result for increased inhibition shown in Fig. 7C, showing response similarity is also decreased in this case. B: Mean similarity over 10 trials at 0 degree orientation for different values of inhibition. Maximal noise robustness is obtained at an intermediate level of inhibition.

Increased inhibition results in better discrimination performance in the case of zero noise (Fig. 8). Fig. 11A shows that this increased performance comes at the cost of decreased noise robustness. It shows, together with the results of Fig. 9B, results for the decreased and increased inhibition columnar network of Fig 7. Decreasing inhibition decreased noise robustness because activity propagates laterally to neighboring “columns” (Fig. 7B). However, increasing inhibition also decreased noise robustness, likely because not enough cells were able to fire in order to reconstitute the correct ensemble. Thus, inhibitory strength has an “inverted U” function (Fig. 11B) reflecting a balance between signal discrimination and signal recovery in the face of noise [48]. This nonmonotonic dependency of performance on network excitability has also been demonstrated for serotonin [49].

### Synaptic failures

Our model supports the conclusions of Song et al. [50], in that the network is comprised of a sea of weak connections (between pyramidal cells) with a small number of stronger connections embedded (their Fig. 9, our Fig. 4C-E). The strong connections are primarily responsible for the coherent activation of the cell ensemble most appropriate to the stimulus. The weaker connections, although numerically far superior, contribute little to this function. We hypothesized that the phenomenon of synaptic failures may act to mitigate this discrepancy.

**Fig 12:**
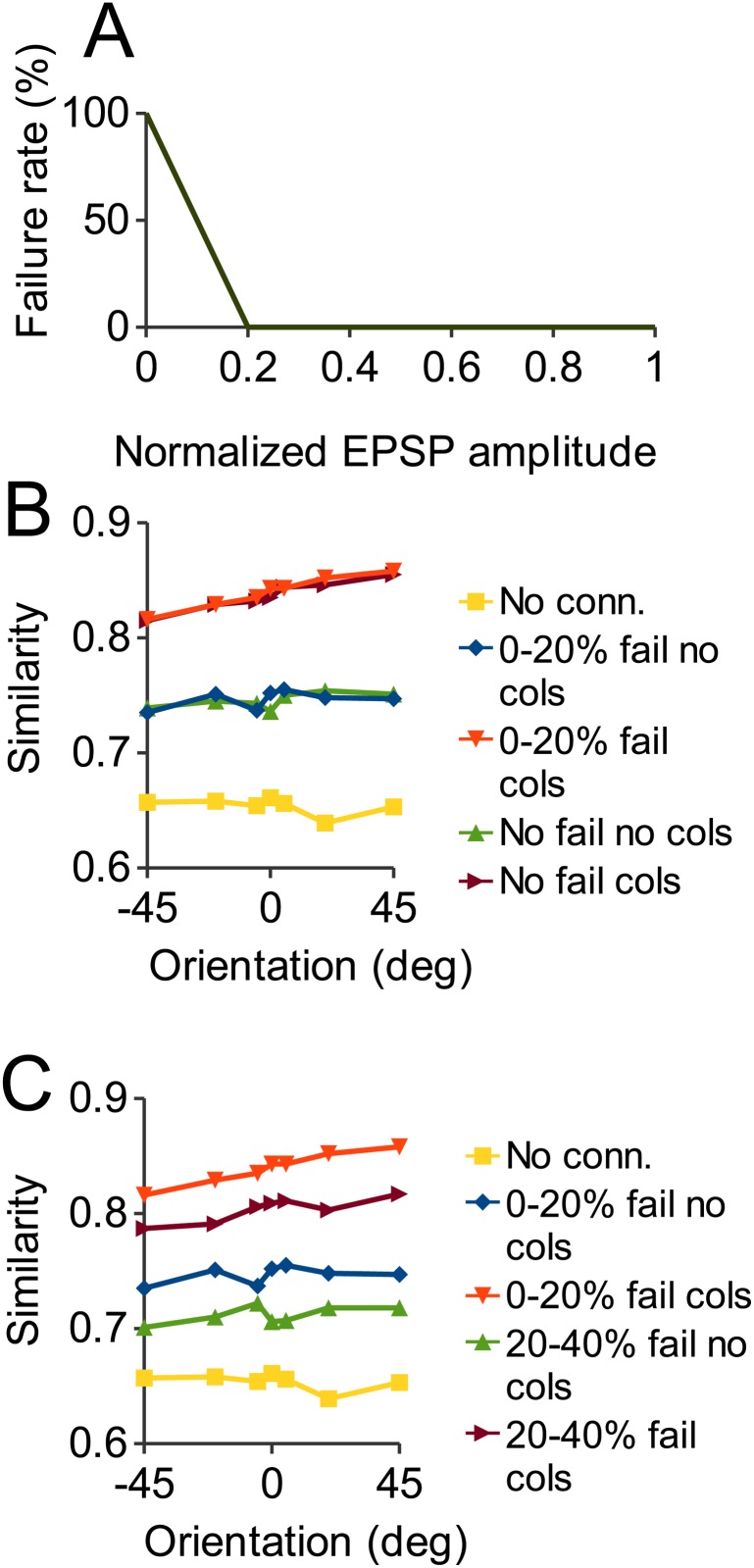
Effect of synaptic failures on network noise robustness. A: Piecewise linear function used as approximate fit to experimental data showing dependence of synaptic failure rate on synaptic strength. Synapses with a strength above 20% of maximum do not fail. Below this, failure rate is an inverse linear function of strength. B: Response similarity as in Fig. 9B, showing the results with default synaptic failure (as in A).”No fail” plots are results when all synapses are reliable, i.e. the line in Fig. 12A is flat at zero. Even though the addition of extra functional synapses increases the firing of the network, there is no increase in noise robustness. C: As a control, instead of the weakest 20% of synapses, failure applies to synapses with strengths 20–40% of the maximum. Weakening these synapses does result in an impairment of noise robustness, indicating synapses with these strengths do make a functional contribution.

Synapses between pyramidal cells in mammalian primary sensory cortex are reliable (presynaptic spike always gives rise to postsynaptic EPSP) except when the amplitude of the EPSP is small [16], [40]. The unreliability of weak synapses effectively reduces the mean EPSP strength of these synapses. All results above include the correction for synaptic failures shown in Fig. 12A (see Methods). Without this correction (i.e. all synapses fully functional) the number of spikes fired by the network in response to stimulation increased (by 8.4% for the columnar network and 9.4% for the non-columnar case). However, Fig. 12B shows that the addition of these extra spikes contributed nothing to the noise robustness of the network. Thus, the synapses that tend to fail to release transmitter are precisely those that do not contribute to performance in a noise robustness test. In our model synapses are weak because they are not part of a strongly interconnected ensemble; they are typically connections between cells with different stimulus preferences. Therefore, it may be that synaptic failure is a way to conserve metabolic resources by reducing transmitter release at synapses that currently do not contribute to network function. 43.5% of synapses had strengths in the bottom 20% of the distribution and 1.6% in the top 20% (see also Fig. 4C). The control (Fig. 12C; see Methods) shows that it is possible to affect performance with synaptic failures, with the failure of medium strength synapses significantly degrading the noise robustness of the non-columnar and columnar networks.

## Discussion

### Functional significance of columnar architecture

The main result of this investigation is that columnar structure improves the noise robustness of cortical networks (Fig. 9). This is because pyramidal cells in layer 2/3 preferentially connect to others with similar stimulus parameter tuning [17]. Therefore, when cells with similar tuning are physically close to each other (i.e. columnar organization) they will have more appropriate targets and consequently form more densely connected and stronger ensembles (Fig. 4). Conversely, in a non-columnar cortex there will be cells that are very close in tuning, therefore connecting them would support the robustness of their ensemble, but they are physically too far apart because of the “salt and pepper” distribution [22] and the dependence of connection probability on distance [15], [16]. Although no differences between columnar and non-columnar cortices have been reported when considering single cell properties such as orientation tuning or behavioral performance such as visual acuity [7], we predict a difference in orientation discrimination, considering both time taken to converge on maximal performance (Fig. 9C) as well as the relative levels of maximal performance in the face of visual noise. This should be tested in equivalent animals, both visual with approximately the same size V1, for example squirrels (non-columnar) vs. minks or ferrets (columnar). In addition, the model predicts that columnar animals should show an EPSP amplitude distribution that is more log-normal than the near exponential distribution shown by non-columnar animals (Fig. 4C).

### What is a cortical column?

Traditionally, columns have been considered as discrete modules, perhaps even based on physical clustering of neuronal elements [5], [13]. However, simple topographic sensory mapping such as in, for example, barrel cortex, is not considered a truly cortical columnar phenomenon [8] and mapping has been shown to be continuous down to the single cell level [4], [11]. Additionally, the model shows that the actual ensemble of cells that is activated in response to a particular stimulus is distributed over hundreds of microns (Fig. 5) rather than being composed of a group of immediately adjacent cells, thus “columns”, in as much as they exist, are a continuous and overlapping phenomenon. This continuity reflects the continuous variability of visual features: Any particular combination of visual features will activate a particular ensemble of cells, instantaneously defining a “column” at the mesoscopic map level but the most active cells will be scattered across this column at the single cell level. A change in the parameters of the stimulus will lead to a different ensemble being activated, although ensembles will overlap (contain common members) if the difference between the two stimuli is small (Fig. 2).

We are not denying, of course, the extensive vertical organization of cortical connectivity across layers. This well-documented feature of cortical circuitry, which may be common across areas and species regardless of the presence of columnar organization, could be the basis for a “generic cortical algorithm” applied to all inputs [51], [52].

Because of the shape of the distribution of EPSC strengths (Fig. 4C,E), most synapses will be relatively weak and network activity will be dominated by a small number of strong connections that form tight clusters, as suggested by Song et al. [50]. Weaker synapses make less contribution to network function (Fig. 12) and are more likely to fail [16], [40], perhaps in order to conserve metabolic resources. It may be that a large number of synapses in layer 2/3 are ineffective since they are not currently members of a tightly connected ensemble. However, weak synapses undergo paired-pulse facilitation and strong synapses paired-pulse depression [40], thus firm conclusions await a detailed study of synaptic temporal dynamics. Available evidence also indicates that weaker synapses are readily increased in amplitude [53], indicating that cluster structure can adapt to the changing statistics of the input over time.

It may be more accurate to talk about groups of synapses (rather than groups of cells) as members of ensembles or clusters, since any cell has thousands of synapses, some of which may be strongly potentiated and part of an active ensemble, while the majority of synapses are weaker and not currently involved in firing the cell. The strong synapses that connect the cells firing the most to a particular stimulus best define the ensemble since any particular cell might also fire as a member of other ensembles. It is also possible that potentiating members of a pool of weak synapses may provide a metabolically cheap way to increase “effective connectivity” in a network rather than synthesizing new synapses to provide structural plasticity [54].

### Discrimination vs. noise robustness

Inhibition in the model implements a divisive normalization function [55], in the sense that a uniform inhibition reflects summed excitatory cell activity [33], [35] (see Methods). Due to the way input is modeled there is effectively no input to cells preferring orthogonal orientations to the stimulus, so no need for cross-orientation inhibition. Instead inhibition works on cells with tuning close to that of the stimulus to produce selective sparse output of the pyramidal cell network, in an analogous way to a circuit in the locust mushroom body [56]. The selection occurs as described by Almeida et al. [44]: varying levels of excitatory input (reflecting tuning) drive pyramidal cells towards threshold. The best-tuned cells will fire first, exciting the similarly-tuned members of their ensemble and activating inhibition to suppress less well-tuned pyramidal cells. The inhibition effectively decorrelates, improving the discrimination of similar input signals [57], [58].

The model shows that there is a tradeoff between discrimination and noise robustness (Fig. 11) that depends on the strength of inhibitory feedback. Theoretical results suggest that the optimal population coding strategy depends on the level of noise, with negative coupling (inhibition) between cells best for low noise and positive coupling (excitation) better when noise levels are high [48]. Our model shows that for a fixed level of noise there is an optimal strength of inhibition that provides discrimination performance equal to that of an uncorrelated (unconnected) network while also providing significant robustness to noise.

### Role of intralaminar recurrent connectivity

The columnar network shows significantly increased firing rates when compared to the unconnected network. Due to the specific connectivity of the recurrent excitatory feedback in layer 2/3, the output of cells best tuned to the stimulus is amplified while global feedback inhibition acts to reduce the firing of less well tuned cells. This increases the signal-to-noise ratio, providing noise robustness to the network. Thus, rather than a compensation for weak feedforward inputs [51], amplification is an integral part of the pattern completion function of cortical circuitry [59]. Ko et al. [60] have recently shown that the specific structure of the recurrent connections between layer 2/3 pyramidal cells is established after receptive field formation and they suggest that these connections amplify the signal and contribute to the robustness and reliability of the cortical representation.

This study considers only local connectivity within a single layer in a column. The model cells displayed almost the same tuning as their feedforward inputs, as is seen in layer 4 simple cells [61]. Those layer 4 cells receive most of their (non-feedforward) input from other simple cells within a few hundred microns, as in the architecture of our model. Local connectivity functions to discriminate similar stimuli while stabilizing the representation in the face of noise. The local circuitry preserves feedforward tuning because of the connectivity constraint (distance and feature dependent connectivity), but 50% or more of the synapses received by any L2/3 pyramidal cell originate from cells at a distance of greater than 500–1000 μm [62]. This significant contextual input could cause the tuning of the cells to deviate from that of their feedforward inputs [63–65].

These results support a mnemonic function for cortical circuitry [66–68] in which the “computation” performed is essentially the differentiation and stabilization of a particular pattern previously established through experience. This pattern, defined by feedforward, vertical cortical connections, is reinforced by recurrent, horizontal connections in a form of associative memory [69].
